# Impact of Rh, Ru, and Pd Leads and Contact Topologies on Performance of WSe_2_ FETs: A First Comparative Ab Initio Study

**DOI:** 10.3390/ma17112665

**Published:** 2024-06-01

**Authors:** Chih-Hung Chung, Chiung-Yuan Lin, Hsien-Yang Liu, Shao-En Nian, Yu-Tzu Chen, Cheng-En Tsai

**Affiliations:** Department of Electronics and Electrical Engineering and Institute of Electronics, National Yang Ming Chiao Tung University, Hsinchu 300, Taiwan; h33188h33188@gmail.com (C.-H.C.); hyliu.ee10@nycu.edu.tw (H.-Y.L.); kece96572@gmail.com (S.-E.N.); sally8863@gmail.com (Y.-T.C.); a871103@gmail.com (C.-E.T.)

**Keywords:** non-van der Waals (vdW) sandwich contacts, non-equilibrium Green’s function (NEGF), transition metal dichalcogenide (TMD), WSe_2_/Rh, WSe_2_/Ru, WSe_2_/Pd

## Abstract

2D field-effect transistors (FETs) fabricated with transition metal dichalcogenide (TMD) materials are a potential replacement for the silicon-based CMOS. However, the lack of advancement in p-type contact is also a key factor hindering TMD-based CMOS applications. The less investigated path towards improving electrical characteristics based on contact geometries with low contact resistance (*R*_C_) has also been established. Moreover, finding contact metals to reduce the *R*_C_ is indeed one of the significant challenges in achieving the above goal. Our research provides the first comparative analysis of the three contact configurations for a WSe_2_ monolayer with different noble metals (Rh, Ru, and Pd) by employing ab initio density functional theory (DFT) and non-equilibrium Green’s function (NEGF) methods. From the perspective of the contact topologies, the *R*_C_ and minimum subthreshold slope (*SS*_MIN_) of all the conventional edge contacts are outperformed by the novel non-van der Waals (vdW) sandwich contacts. These non-vdW sandwich contacts reveal that their *R*_C_ values are below 50 Ω∙μm, attributed to the narrow Schottky barrier widths (SBWs) and low Schottky barrier heights (SBHs). Not only are the *R*_C_ values dramatically reduced by such novel contacts, but the *SS*_MIN_ values are lower than 68 mV/dec. The new proposal offers the lowest *R*_C_ and *SS*_MIN_, irrespective of the contact metals. Further considering the metal leads, the WSe_2_/Rh FETs based on the non-vdW sandwich contacts show a meager *R*_C_ value of 33 Ω∙μm and an exceptional *SS*_MIN_ of 63 mV/dec. The two calculated results present the smallest-ever values reported in our study, indicating that the non-vdW sandwich contacts with Rh leads can attain the best-case scenario. In contrast, the symmetric convex edge contacts with Pd leads cause the worst-case degradation, yielding an *R*_C_ value of 213 Ω∙μm and an *SS*_MIN_ value of 95 mV/dec. While all the WSe_2_/Ru FETs exhibit medium performances, the minimal shift in the transfer curves is interestingly advantageous to the circuit operation. Conclusively, the low-*R*_C_ performances and the desirable *SS*_MIN_ values are a combination of the contact geometries and metal leads. This innovation, achieved through noble metal leads in conjunction with the novel contact configurations, paves the way for a TMD-based CMOS with ultra-low *R*_C_ and rapid switching speeds.

## 1. Introduction

With continued transistor downscaling, the short channel effect becomes more notorious and urgently needs alleviation [1,2,3,4,5]. In order to address this challenge, the emerging 2D transition metal dichalcogenides (TMDs) are referred to as promising candidates owing to their exceptional properties [6,7,8,9,10,11], such as a desirable band gap, dangling-bond-free surface, slight mobility variation, and ultra-thin thickness [11,12,13,14,15,16,17,18]. Among these TMDs, WSe_2_ has attracted great interest in p-FETs, with early reports of WSe_2_-based FETs showing hole mobility [19,20,21,22]. These experimental hole mobilities are reported within 100~500 cm^2^/V-s. Also, WSe_2_ has pronounced thermal and environmental stability [23], making it an attractive channel material for TMD-based FETs. 

**Figure 1:**
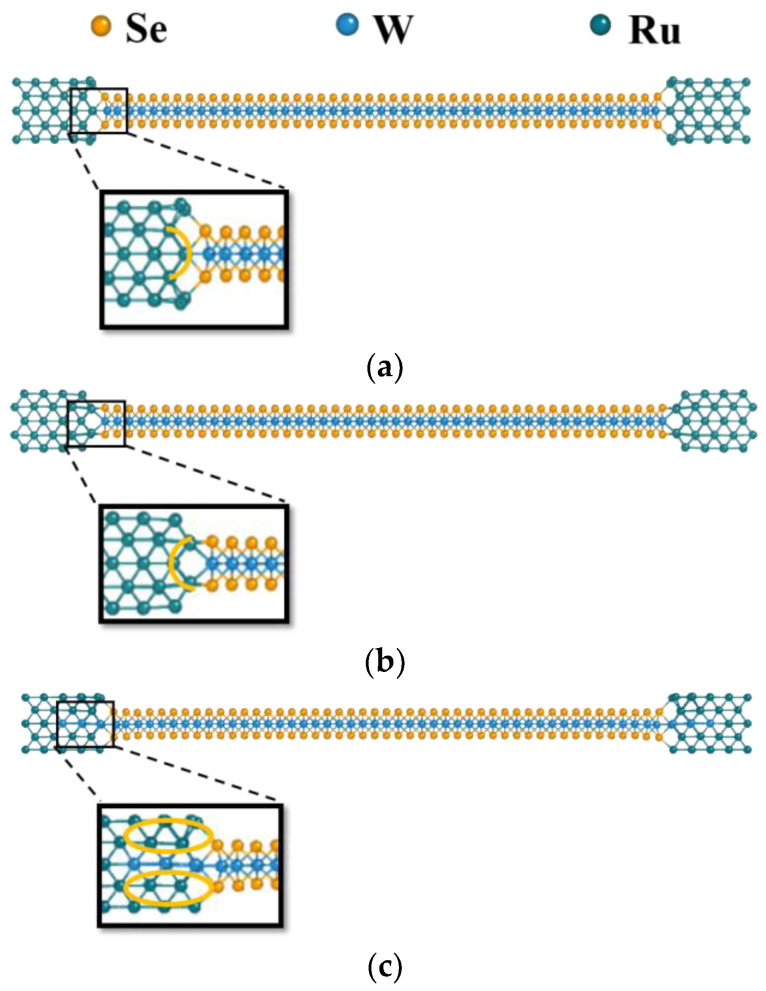
WSe_2_/Ru contacts as an example to illustrate (**a**) the symmetric convex edge contacts, (**b**) the symmetric concave edge contacts, and (**c**) the non-vdW sandwich contacts.

Inevitably, 2D TMD-based FETs have contact interfaces with 3D metal leads [24,25,26,27,28,29,30], serving as source and drain regions. For example, Jacko Rastikian et al. used Co and Pd as contacts to obtain high-performance p-type WSe_2_ FETs [27]. Dae Hyun Jung et al. reported that Ti/Au were deposited as contacts for their monolayer WSe_2_ FETs [29]. Ang-Sheng Chou et al. proposed that Sb-Pt contact engineering can achieve the excellent performance of p- and n-type monolayer WSe_2_ FETs [30]. Fundamentally, contact geometries include top and edge contacts [14,17,31]. Edge-contacted configurations enhance the in-plane carrier injection to the atomically thin TMD and involve no additional tunneling barrier along the current path [14,17]. Thus, such contacts minimize the contact resistance (*R*_C_) compared to top contacts. Moreover, edge-contacted configurations provide optimal device scalability [32] and compatible 3D integration [33]. Based on the advantages mentioned above, edge contacts are adopted for this study. Furthermore, the atomic-scale characterization of the interfaces has garnered interest in research over the past years [34,35,36,37], so the atomistic structural variation across the TMD/metal interfaces is also considered. The atomistic interfaces can be divided into two categories based on their structural variants: symmetric convex and concave edge contacts. The contacts mentioned above are shown and further detailed in Figure 1. The symmetric convex edge contacts mean that the convex surface of the source electrode, indicated by the yellow line in Figure 1a, aligns with the TMD across the interface. Conversely, the symmetric concave counterparts shown in Figure 1b are the opposite. Hence, the discrepancy between these edge contacts can be indicated at the atomistic level, demonstrating the different electrical behaviors. 

Apart from the above-mentioned edge contacts, a novel contact configuration is also being studied. Conventionally, the top contacts can be easily constructed by deposition, meaning the manufacturing challenges are smaller than the edge counterpart but exhibit a van der Waals (vdW) gap between the leads and TMD. Such a vdW gap inevitably leads to an undesirable tunnel barrier, further degrading the carrier injection efficiency across the interfaces [17,38] for Schottky barrier (SB) TMD-based FETs. That is, alleviating tunnel barriers can initially improve the electrical contacts. Yaochen Sheng et al. have demonstrated that contact behaviors can be improved by removing the facial chalcogen atoms of the TMD to eliminate the tunneling barrier induced by the vdW gap through direct contact with metal leads [39]. As shown in the previous work [39], the source/drain of the TMD can be patterned after lithography and development. Next, the defined TMD is then treated by H_2_ plasma etching. The upper chalcogen atoms are thus removed after the weakest-ion bombardment. After metal deposition, direct metal contact with the TMD can be formed at the source/drain regions, which are expected to improve the *R*_C_. Undoubtedly, these manufacturing techniques and steps are feasible so far, and the above-mentioned process is subsequently generalized in our work to create a novel contact topology. Our primary focus is combining edge and direct top contact, allowing both elements to employ their strengths to obtain optimized contact performances. Thus, partial selenium replacement for both sides of a TMD is critical to achieving novel contact engineering. In the beginning, the edge-contacted interfaces are fabricated in a conventional process. Second, the upper side of the TMD has to be patterned to define source/drain “extension” regions near the TMD/metal interfaces, compared to the symmetric convex edge contacts. Such regions then undergo H_2_ bombardment, as previously mentioned. Successively, the upper chalcogen atoms near the TMD/metal interfaces are replaced with those of the edge-contacted metal leads by metal deposition. However, the adoption of such contacts is not without any obstacles. The non-vdW contacts have partly been formed so far, and the subsequent difficulties have added layers of complexity to fabrication. Substituting the chalcogen atoms of the bottom layer with Rh, Ru, or Pd adds challenges to the sample fabrication. The hurdles inevitably lie in flipping the semi-finished device upside down, without causing any defects, to continue the successive processing steps. After that, the original bottom layer of the device can be further processed to complete the non-vdW sandwich contacts. Repeating patterning and deposition, as mentioned above, can replace these chalcogen atoms with metal lead counterparts at the remaining source/drain “extension” regions. Due to meticulous “flip” treatments, the relevant processes have remained technically unfeasible and have not yet been experimentally realized. Nevertheless, semiconductor technology is advancing quickly, so the realization of such a novel contact configuration can be expected. As highlighted by two yellow ellipses in Figure 1c, the so-called non-vdW sandwich contacts can be thus proposed through high manufacturing complexity. Although non-vdW sandwich contact engineering requires a highly sophisticated and complex process for existing techniques, our simulated results can shed light on developing such a contact-engineered FET at the initial state. 

In addition to these contact geometries, noble metals, as the contact leads, play a crucial role in achieving extraordinary nanomaterial-based FETs [40]. For example, Rh is prone to make near-Ohmic contacts with single-walled carbon nanotube field-effect transistors (CNTFETs) [40]. For scaled edge-contacted CNTFETs, the *R*_C_ exhibits better scaling behaviors by decreasing the contact length of the Rh leads [41]. For micro-electromechanical systems (MEMSs), Rh emerges as a highly ranked contact metal, considering its high melting point and low *R*_C_. Hence, the idea of using Rh is generalized in our study to form WSe_2_/Rh contacts and is anticipated to improve the electrical characteristics of p-type WSe_2_ FETs. Additionally, the scientific community has traditionally used Pd as the contact metal [42,43,44,45,46]. The high work function of Pd is 5.2 eV [47], which thus facilitates the noble metal to inject and conduct the holes into the WSe_2_ channel through the suitable Fermi level (*E_F_*) alignment of the metal leads with the edge of the valence band in the TMD. Patoary et al. proposed that model calculations can achieve an approximately ideal *SS* value and a larger *I*_ON_ of ~270 µA/µm [48]. Therefore, WSe_2_/Pd contacts are also constructed in conjunction with the various contact topologies and extensively studied in our research. In addition to the contact metals mentioned above, Ru is also an attractive contact metal for next-generation interconnects [49,50,51]. This metal can reduce the resistivity size effect and electromigration [52,53,54,55]. Also, MEMSs have utilized low-resistance Ru to improve contact behaviors [56,57]. These theoretical and experimental results suggest that Ru has a vast potential for excellent electrical contacts. In the TMD-based FET community, O’Brien et al. experimentally reported the low *R*_C_ of WSe_2_ FETs with Ru contacts, accompanied by an on-state current (*I*_ON_) of 50 μA/μm and subthreshold swing (*SS*) of 141 mV/dec [58]. Due to these intriguing properties, WSe_2_/Ru contacts have become a rising star and are worth further study. Although the above-published works indicate excellent p-type performances, there is still a puzzle about the emphasis on the quantum transport concerning the *R*_C_ and contacted configurations. Therefore, it is urgent to establish the optimal combination involving contact metals and their respective configurations.

In this research, our attention is directed toward the novel variants of contact configurations and noble metals. DFT + NEGF calculations are conducted to study the electrical characteristics of WSe_2_/metal (Rh, Ru, Pd) in the different contact-engineered configurations. Our findings unveil that the Schottky barrier widths (SBWs) and Schottky barrier heights (SBHs) are extremely sensitive to contact geometries and noble metals. The lowest *R*_C_ and nearly ideal *SS* value can be achieved by the non-vdW sandwich WSe_2_/Rh FETs, further providing theoretical support for the design of ultra-scaled TMD-based FETs.

## 2. Materials and Methods

### 2.1. DFT Simulations

The structural relaxations for WSe_2_/metal (Rh, Ru, and Pd) contacts are performed based on density functional theory (DFT) using the exchange-correlation potential of local density approximation (LDA) and projector augmented wave (PAW) pseudopotentials [59], as implemented in Vienna ab initio Simulation Package (VASP) [60,61]. One 16 Å vacuum layer is set along the vertical direction to avoid the spurious interaction between adjacent repeating cells. The cutoff energy of 400 eV is used for the plane wave expansion. A k-point mesh of 1 × 10 × 1 is adopted in the reciprocal lattice. For the WSe_2_ monolayer contacted to the metal leads, the rectangular surfaces (110) of the metal leads are adopted to fit WSe_2_ with a sufficiently low interfacial strain below 7%. The transport direction is along the *x*-direction, while in the *y*-direction, periodic boundary conditions are employed to represent an infinite WSe_2_ monolayer and two metal leads. During the structural relaxation, the atomic *z*-coordinates of the Se atoms away from the WSe_2_/metal interfaces are fixed. The stopping criterion for the ionic relaxations in these contact structures with different contact geometries is the remnant force on each atom below 0.05 eV/Å, and the convergence criterion for electronic iterations is 10^−4^ eV.

### 2.2. Device Structures and Transport Simulations

**Figure 2:**
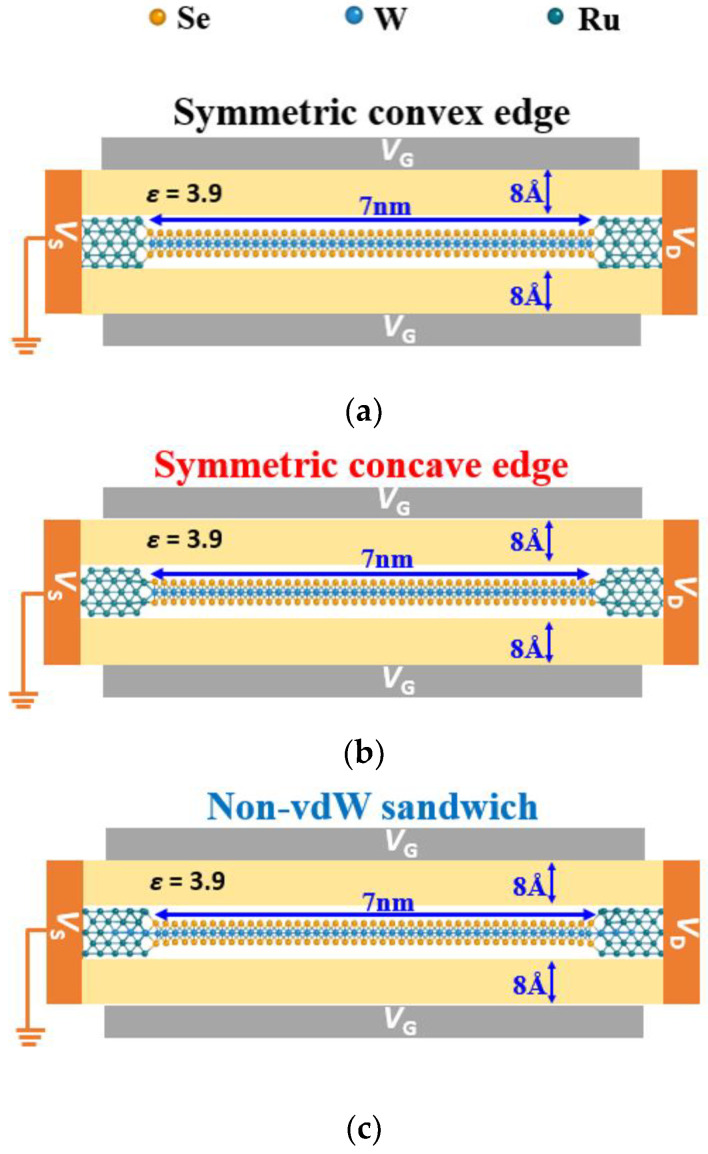
WSe_2_/Ru contacts as an example to illustrate a simulated double-gate monolayer TMD-based FET with a 7 nm channel. The gate oxide is set to be 8 Å. (**a**) Symmetric convex edge contacts. (**b**) Symmetric concave edge contacts. (**c**) Non-vdW sandwich contacts.

All schematic views of the simulated double-gate device structures are shown in Figure 2 and generated using the NEGF-DFT NanoDCAL package [62,63,64]. NanoDCAL employs the double-zeta-polarized (DZP) atomic orbital basis set to extend all physical quantities. The standard norm-conserving pseudopotential defines the atomic cores [65]. LDA is chosen for the exchange-correlation functional. The truncation energy of the self-consistent atomic orbital is set to 80 Hartree. The convergence criterion of the density and Hamiltonian matrices in the self-consistent calculations is set to 10^−4^ eV. Spin-orbit coupling (SOC) and the temperature of 300 K are both considered in the theoretical simulations. The channel length (*L*) equals 7 nm, and the gate length is set to 9.8 nm. The device structure is periodic in the channel width direction. The k-point mesh in the self-consistent calculation is set to 1 × 10 × 1 for the central scattering regions. A 1 × 100 × 1 k-grid is used to calculate the transport current. The equivalent oxide thickness (EOT) is 8 Å, and the dielectric constant is 3.9. The drain-to-source voltage (*V*_DS_) is applied at −50 mV [66] to allow the drain current (*I*_D_) to be determined by the nature of the SB.

## 3. Results and Discussion

### 3.1. Transport Properties

**Figure 3:**
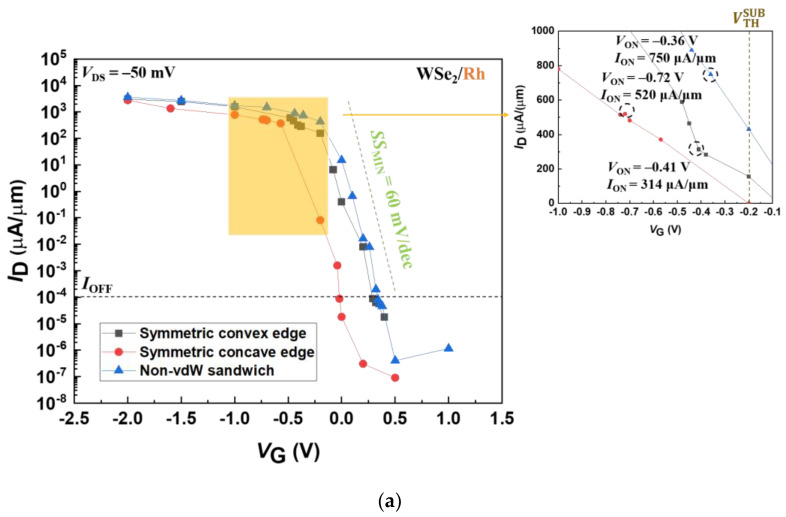

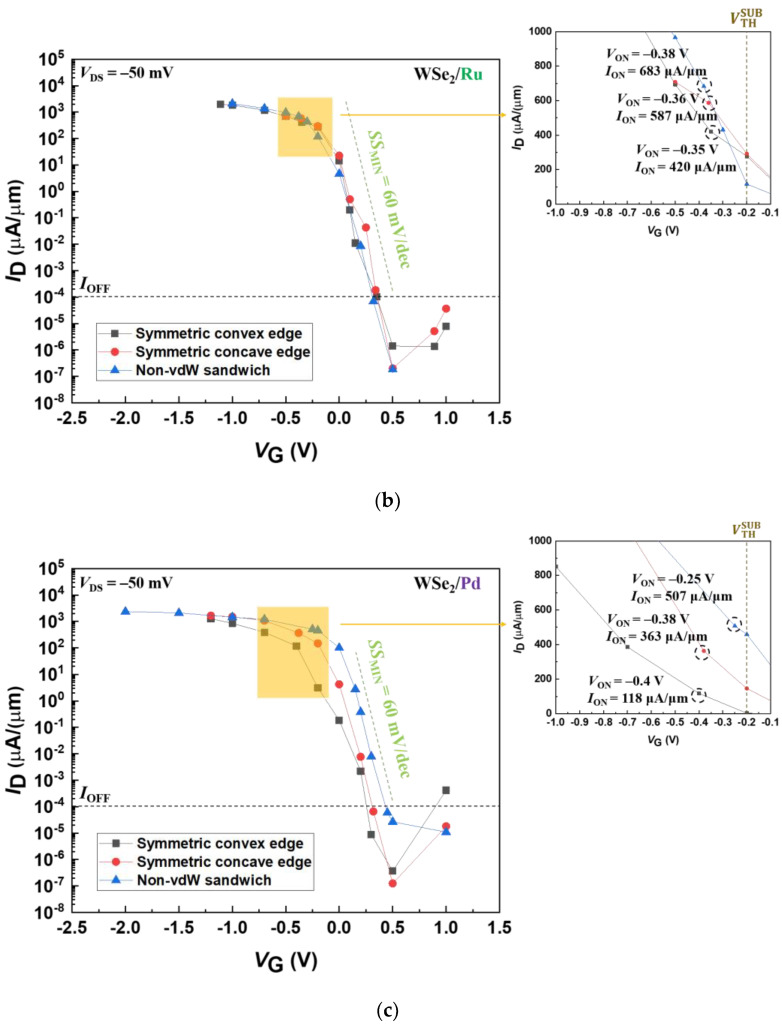
*I*_D_−*V*_G_ curves of WSe_2_ FETs with (**a**) Rh, (**b**) Ru, and (**c**) Pd as metal leads based on three different contact configurations. Inset: Zoom in on the data of *I*_ON_ and *V*_ON_ in the on states, indicated by dashed circles. *V*_G_ is the voltage applied to the gate electrode. *I*_D_ is when the direct current enters the drain electrode with a specified *V*_G_. *V*_ON_ refers to the on-state gate voltage when *V*_G_ is in the off state plus a negative voltage of −0.7 V. *I*_ON_ is *I*_D_ when the MOSFET is biased to the on state. $V_{TH}^{SUB}$ represents the *V*_G_ in the subthreshold regime. *V*_DS_ is set to −50 mV.

Past work reported by Jung et al. [67] shows that the best sheet resistance (*R*_SH_) of WSe_2_, which has less than ten layers, maintains about 10^3^ Ohm/sq. Given that our simulated 2D FETs do not include electron-phonon scattering and defects, the WSe_2_ monolayer can be a few Ω∙μm. Hence, such a relatively low *R*_SH_ makes the *R*_C_ dominate the resistance of the whole system. To access the *R*_C_ of these various contact-engineered WSe_2_/metal FETs, the *I*_ON_ among the various WSe_2_/metal contact geometries is first determined. Herein, the *I*_ON_ is the *I*_D_ when the difference between the gate voltage (*V*_G_) at the off-state current (*I*_OFF_) level and the turn-on voltage (*V*_ON_) reaches 0.7 V, i.e., |*V*_G_ − *V*_ON_| = 0.7 V [68]. Also, *I*_OFF_ is defined as 10^−4^ μA/μm, represented by the horizontal dashed lines in Figure 3. Additionally, a subthreshold voltage ($V_{TH}^{SUB}$) of −0.2 V is referred to as a representative *V*_G_ in the subthreshold region and will be discussed in more detail later. Apparently, not only are these on/off current ratios larger than 10^6^, but their corresponding high *I*_ON_ values are within the range of 118 μA/μm to 750 μA/μm in the insets of Figure 3a–c. These notably surpass the previous works, making one confident in further studying these three structural variants.

To look closer at the effects of the different contact configurations on their electrical behaviors, we also consider the minimum subthreshold swing (*SS*_MIN_). These computed results, shown in Table 1, are further compared below. Regardless of the contact leads, the non-vdW sandwich contacts have the highest *I*_ON_ levels (507~750 μA/μm), followed by the symmetric concave edge contacts (363~587 μA/μm) and their symmetric convex counterparts (118~420 μA/μm). For the *R*_C_, the opposite thus applies to these contact geometries. The lowest *R*_C_ levels reach as low as 33 to 49 Ω∙μm for the non-vdW sandwich contacts. The second lowest levels increase to 42~69 Ω∙μm for the symmetric concave contacts, followed by the highest levels of 60~213 Ω∙μm for their convex counterparts. The above-calculated results suggest that the non-vdW sandwich contacts are superior to the others. Also, considering the steep switching characteristics, the non-vdW sandwich contacts exhibit the fastest switching rate. Table 1 shows the contacts above with the *SS*_MIN_ values from 63 to 67 mV/dec. The symmetric concave edge contacts are ranked second fastest, ranging from 67 to 74 mV/dec, followed by their convex counterparts (81~95 mV/dec). Based on these results, non-vdW sandwich contacts achieve optimal device performances in terms of *R*_C_ and *SS*_MIN_. 

The contact leads can also affect the performances of TMD-based FETs due to the various SBs across the TMD/metal interfaces [69,70]. Through a deep dive into comparing a contact topology with different contact metals, further analysis can be performed by revisiting Figure 3. First, the WSe_2_/Rh FETs generate more shifts in the *I*_D_−*V*_G_ curves than the WSe_2_/Pd FETs. The former shows approximately 500 mV and the latter 250 mV. Furthermore, compared with the above two FETs, the contact-engineered WSe_2_/Ru counterparts exhibit minimal shifts (~50 mV). Via the insets of Figure 3a–c, the shifts of *V*_ON_ clearly show high consistency with those of the transfer characteristics. These *V*_ON_ values shift slightly due to the insubstantial shifts among the corresponding *I*_D_−*V*_G_ curves, and vice versa. This suggests that WSe_2_/Ru FETs can achieve the most stable circuit operation [71], outperforming their WSe_2_/Rh and WSe_2_/Pd counterparts. 

### 3.2. Band Diagrams

**Figure 4:**
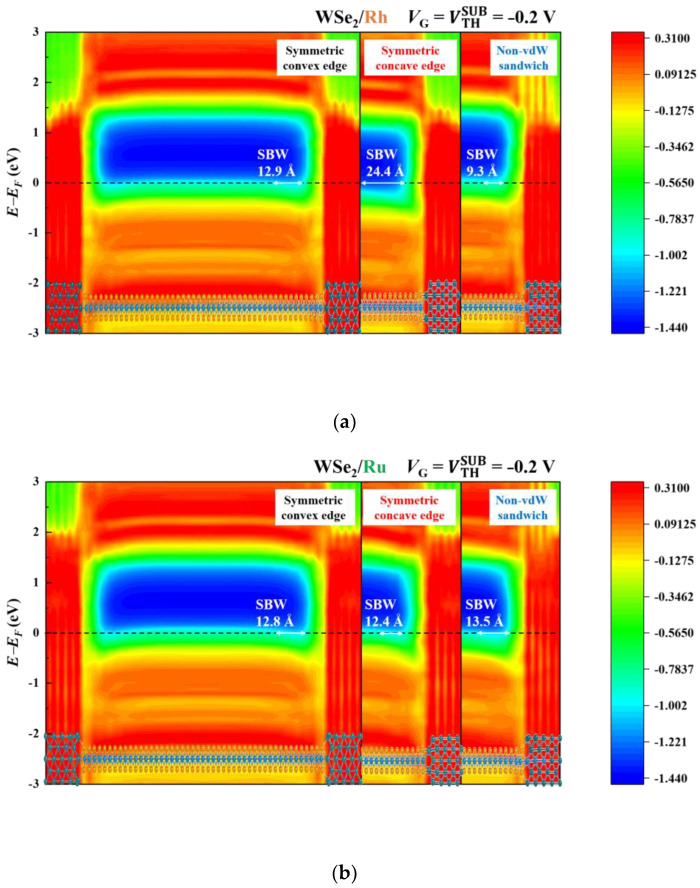

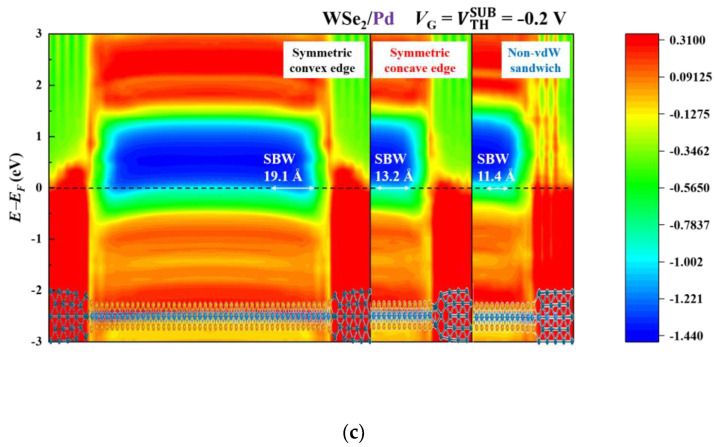
LDDOS of WSe_2_/metal based on the symmetric convex edge, symmetric concave edge, and non-vdW sandwich contacts at *V*_G_ = $V_{TH}^{SUB}$ = −0.2 V and *V*_DS_ = −50 mV. (**a**) WSe_2_/Rh. (**b**) WSe_2_/Ru. (**c**) WSe_2_/Pd.

**Figure 5:**
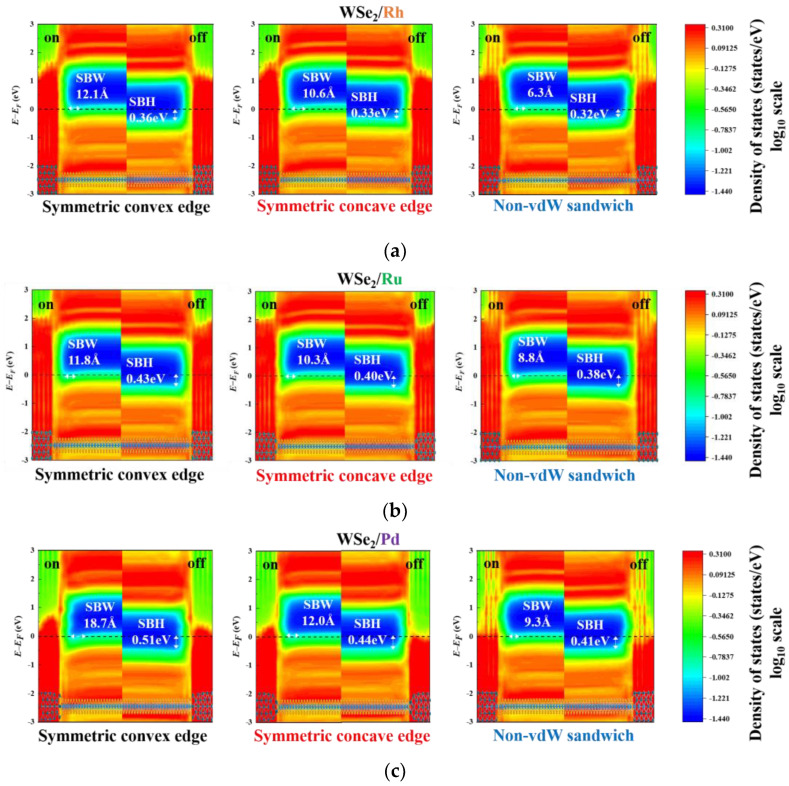
LDDOS of the contacts with a seven nm−long WSe_2_ channel before and after applying *V*_ON_, accompanied by *V*_DS_ = −50 mV (**a**) WSe_2_/Rh. (**b**) WSe_2_/Ru. (**c**) WSe_2_/Pd.

SBWs and SBHs across the TMD/metal interfaces are well-known as critical factors for dominating the performances of the TMD-based FETs [68]. Such two quantities can be indicated by the band diagrams revealed by the local device density of states (LDDOS), further impacting quantum transport. At room temperature, current transport is mainly limited by an SBW when not overcoming an SBH thoroughly. Such a primary consideration is essential when the FET is not turned on. At this stage, *V*_G_ = $V_{TH}^{SUB}$ = −0.2 V is taken to be an example. The corresponding LDDOS for these contact-engineered FETs are illustrated in Figure 4. From Figure 4a, the WSe_2_/Rh FET based on the symmetric concave edge contacts displays the widest SBW of 24.4 Å, compared to the other two contact geometries. This widest SBW suggests that such a WSe_2_/Rh contact configuration offers the lowest *I*_D_. The current flow is lower than the others by four orders of magnitude, as observed in Figure 3a. Next, no significant SBW difference (12.4~13.5 Å) among the three WSe_2_/Ru contact topologies is shown in Figure 4b, although the non-vdW sandwich contacts form the widest SBW. Thus, no considerably different transfer characteristics are established in Figure 3b. For the WSe_2_ contacts with the Pd leads, the symmetric convex edge contacts exhibit the widest SBW (19.1 Å) in Figure 4c, accompanied by the lowest *I*_D_ (a few μA/μm) in Figure 3c. When the applied *V*_G_ reaches *V*_ON_, the FET is then turned on. The simulated WSe_2_ FETs in the on states are displayed in the left panels of each band diagram, as shown in Figure 5. In contrast, the corresponding off states are seen in the right panels. The SBWs and SBHs are depicted on each diagram’s left and right sides. For a single WSe_2_ FET, a significant difference exists among these contact geometries. Take the WSe_2_/Ru contacts in Figure 5b as an example. These undesirable SBHs appear over the range of 0.38 to 0.43 eV, and their corresponding SBWs vary from 8.8 Å to 11.8 Å. Direct and thermally assisted tunneling behaviors are involved in carrier transport across an SB at 300 K. In the on state, the non-vdW sandwich WSe_2_/Ru FET has the narrowest SBW of 8.8 Å and the lowest SBH of 0.38 eV. Consequently, this simulated FET achieves the highest *I*_ON_ of 683 μA/μm due to the minimal SBH and SBW, implying the lowest *R*_C_ value. Next, the SBW is 10.3 Å for the symmetric concave edge contacts, which is more significant than the above-mentioned counterpart, thus having the second-best *I*_ON_ of 587 μA/μm. Lastly, the *I*_ON_ of 420 μA/μm flows through the symmetric convex edge contacts with the highest SBH of 0.43 eV and the widest SBW of 11.8 Å. 

**Figure 6:**
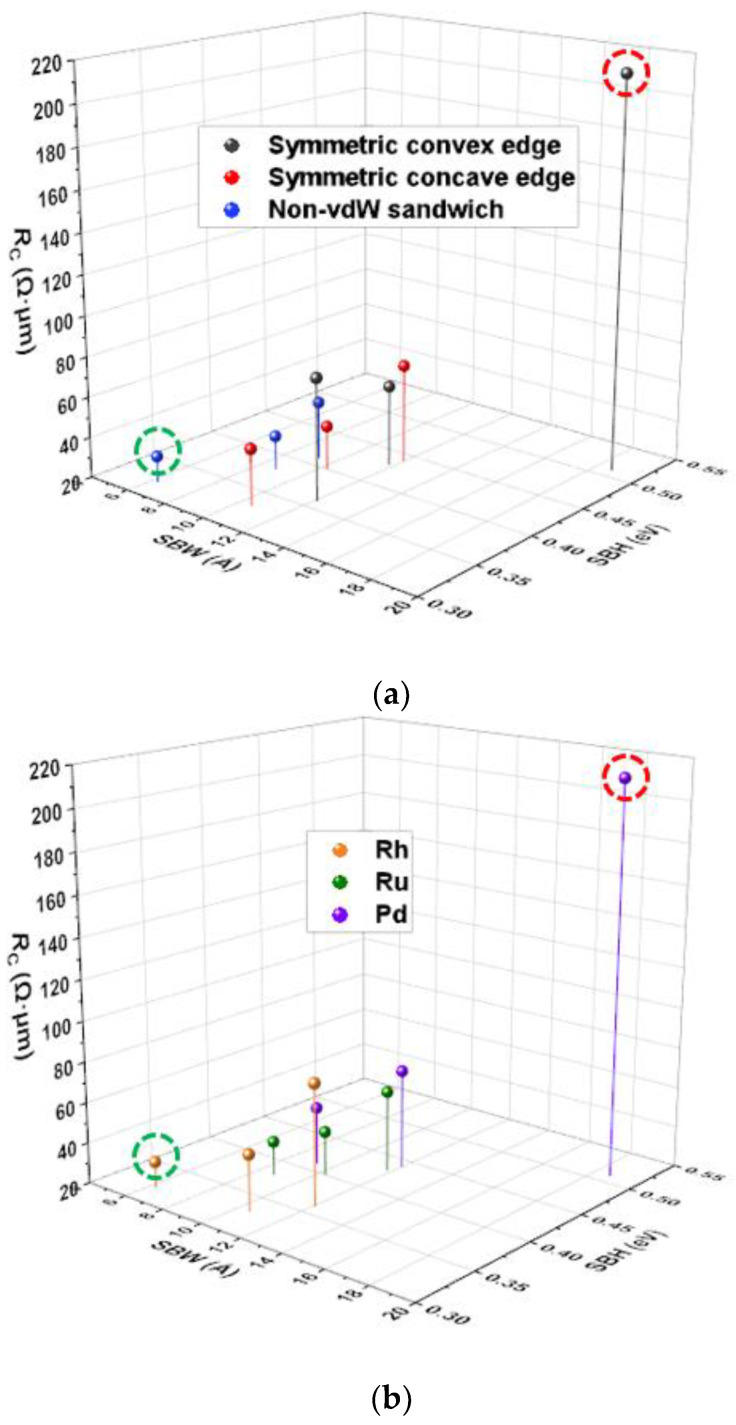
3D scatter plot showing the effects of SBHs and SBWs on *R*_C_ with the category labels of the (**a**) contact geometries and (**b**) contact metals. Dashed green circle: Rh + non-vdW sandwich contacts offers the lowest *R*_C_ with the smallest SBW and SBH. Dashed red circle: Pd + symmetric convex edge contacts displays the highest *R*_C_ with the largest SBW and SBH.

These SB FETs display various electrical properties through all the above contact-engineered geometries. The current can be increased by decreasing the SBW due to the corresponding tunneling probability. Also, the holes with energies above the SBH can be injected into the TMD channel. After conversion to *R*_C_ from *I*_D_ at *V*_DS_ = −50 mV, the above *R*_C_ values are categorized into contact geometries and contact leads, as shown in Figure 6a,b. From the comparison of these nine WSe_2_/metal FETs, the change in *R*_C_ is interestingly consistent with the trend of the contact-engineered SBWs. As depicted in Figure 6a, the non-vdW sandwich contacts (marked in blue) tend to yield a narrower SBW than their symmetric counterparts (marked in red for concave and black for convex, respectively). The data shown in Figure 6b further suggest that Rh (orange) and Ru (green) offer a narrower SBW than Pd (purple). What is even worse is that the higher SBH is formed at various contacted-engineered WSe_2_/Pd interfaces compared to other corresponding interfaces constructed by Ru and Pd. Thus, the corresponding *I*_ON_ is partly hindered by the SBH, therefore deteriorating the contact performances of these WSe_2_/Pd contacts. Finally, the best- and worst-case scenarios are obtained considering the combination of the contact geometries and leads. The WSe_2_/Rh non-vdW sandwich contacts exhibit the lowest SBH of 0.32 eV, whereas their WSe_2_/Pd symmetric convex counterparts display the most undesirable SBH above 0.5 eV. Therefore, the former is expected to have a negligible SBH by applying *V*_ON_, and the latter has a non-negligible SBH at *V*_ON_. Consequently, the WSe_2_/Rh non-vdW sandwich contacts display the lowest *R*_C_ of 33 Ω∙μm, whereas their WSe_2_/Pd symmetric convex edge counterparts show the highest *R*_C_ of 213 Ω∙μm. Conclusively, our demonstration provides a novel strategy to achieve the lowest *R*_C_ and *SS*_MIN_, unveiling the advantages of noble metals over conventional metals. Combining the effects of noble metals and contact-engineered topologies establishes the optimized electrical performances of WSe_2_ FETs. Our optimization serves as a reasonable starting point for fabricating such ultra-scaled FETs. 

## 4. Conclusions

The first comparative study presents the electrical performances of the different contact-engineered WSe_2_ FETs with various noble metal leads. Regardless of the contact metal, the novel non-vdW sandwich contacts improve the *I*_ON_, *R*_C_, and *SS*_MIN_ to the greatest extent. The symmetric concave edge contacts exhibit middling performances, followed by their symmetric convex counterparts. Additionally, the contact metals have quite an impact on the electrical performances of the WSe_2_ FETs. The WSe_2_/Rh FETs are first unveiled, showing the distinguishable *R*_C_ value of 33 Ω∙μm and the nearly ideal *SS*_MIN_ of 63 mV/dec for the non-vdW sandwich contacts. When viewed from another angle, although the Rh leads can lead to the lowest *R*_C_ and *SS*_MIN_, the Ru leads offer minor instability in the circuit in terms of the minimal *I*_D_-*V*_G_ shifts. Moreover, such WSe_2_/Ru FETs exhibit significantly small *R*_C_ (37 Ω∙μm) and *SS*_MIN_ (67 mV/dec) values in non-vdW sandwich contacts. These initial results allow us to evaluate the performances of the TMD-based CMOS from a different perspective. Eventually, the WSe_2_/Pd FETs based on the non-vdW sandwich contacts yield a slightly higher *R*_C_ (49 Ω∙μm) and lower *SS*_MIN_ (64 mV/dec) than the WSe_2_/Ru FETs. However, such WSe_2_/Pd FETs formed by the symmetric convex contacts cause the degradation of *R*_C_ and *SS*_MIN_, increasing *R*_C_ and *SS*_MIN_ up to 213 Ω∙μm and 95 mV/dec. The corresponding performance deterioration in *R*_C_ is approximately 4.5 times higher, while *SS*_MIN_ increases by almost 1.5 times. Furthermore, the above-mentioned results enable a more comprehensive look at the TMD-based FETs from an atomistic perspective. In practice, the edge contact interface exhibits a mixture of convex and concave constituents after fabrication. Therefore, the overall electrical performance is affected by these two components. By choosing suitable noble metal leads, the edge-contacted WSe_2_ FETs have an *R*_C_ within 42~213 Ω∙μm, accompanied by an *SS*_MIN_ from 67 to 95 mV/dec. The rapidly advancing technology can finally realize the proposed non-vdW sandwich contacts. This non-vdW novelty theoretically maintains an *R*_C_ at a low level of 33~49 Ω∙μm, outperforming the edge-contacted configurations. Additionally, the novelty has a faster switching transition. Their *SS*_MIN_ values range from 63 to 67 mV/dec, superior to the edge-contacted topologies. Our simulations display excellent potential for high-performance p-type FETs, paving the way for a future TMD-based CMOS with a low *R*_C_ and high switch speed during the off/on transition. 

## Figures and Tables

**Table 1:** Summary of the critical electrical performances of the simulated TMD-based FETs in this work. *I*_ON_ (μA/μm) is the on-state current. *R*_C_ (Ω∙μm) is the contact resistance. *SS*_MIN_ (mV/dec) refers to the minimum subthreshold swing.

Contact Metal	Contact Geometry	*I*_ON_	*R*_C_	*SS*_MIN_
Rh	Symmetric convex edge	314	80	82
	Symmetric concave edge	520	48	70
	Non-vdW sandwich	750	33	63
Ru	Symmetric convex edge	420	60	81
	Symmetric concave edge	587	42	74
	Non-vdW sandwich	683	37	67
Pd	Symmetric convex edge	118	213	95
	Symmetric concave edge	363	69	67
	Non-vdW sandwich	507	49	64

## Data Availability

The data in this study can be provided upon request.
